# To: Goal-directed therapy guided by the FloTrac sensor in major surgery: a systematic review and meta-analysis

**DOI:** 10.62675/2965-2774.20240168-en

**Published:** 2024-11-26

**Authors:** Luigi La Via, Giacomo Cusumano, Christian Zanza, Carmelo Calvagna, Antonino Maniaci

**Affiliations:** 1 Azienda Ospedaliero Universitaria Policlinico Department of Anaesthesia and Intensive Care Catania Italy Department of Anaesthesia and Intensive Care, Azienda Ospedaliero Universitaria Policlinico "G. Rodolico - San Marco" - Catania, Italy.; 2 University of Catania Department of Surgery and Medical-Surgical Specialties Catania Italy Department of Surgery and Medical-Surgical Specialties, University of Catania -Catania, Italy.; 3 University of Rome Residency Program in Geriatric Medicine Rome Italy Residency Program in Geriatric Medicine, University of Rome "Tor Vergata" -Rome, Italy.; 4 University of Pittsburgh Department of Anesthesiology and Perioperative Medicine Pittsburgh United States Department of Anesthesiology and Perioperative Medicine, University of Pittsburgh - Pittsburgh, United States.; 5 University "Kore" Faculty of Medicine and Surgery Enna Italy Faculty of Medicine and Surgery, University "Kore" - Enna, Italy.

Dear Editor,

We read with great interest the meta-analysis by Alves et al.,^(1)^who highlighted the effects of goal-directed therapy (GDT) guided by the FloTrac sensor in patients undergoing major surgery. This meta-analysis of randomized controlled trials is relevant and confirms a possibly preferential role for GDT in this population of patients, considering the significant improvement in clinical outcomes, reduced hospital and intensive care unit (ICU) stays and reduced mechanical ventilation time. These findings align with the current literature since minimally invasive hemodynamic monitoring has recently shown potential in guiding fluid therapy both in the operating theatre and in the ICU.^(2,3)^

However, before drawing firm conclusions on the role of GDT in patients undergoing major surgery, an analysis of the robustness of the findings provided by Alves et al.^(1)^ is needed. Therefore, we think the manuscript would greatly benefit from the addition of trial-sequential analyses (TSAs), which would allow us to calculate the required sample ("information size"), estimate the power of the meta-analysis on the investigated outcomes, as well as the need for further research.

Thus, we would like to offer such a contribution. We imported the same data provided by the authors into the TSA Software (Copenhagen Trial Unit's TSA Software^®^; Copenhagen, Denmark). The information size was computed assuming an alpha risk of 5% with a power of 80%. The estimated mortality was computed via weighted averages from the included studies. We used a random-effects model, with outcomes analyzed as relative risk or mean difference. Further details on the TSA and its interpretation are available elsewhere.^(4,5)^

The TSAs revealed that current evidence is severely underpowered to determine whether GDT reduces mortality compared with usual care. Similarly, the ratios between the number of patients recruited and the sample needed for the risk of myocardial infarction and hypotension were n = 1172/5415 and n = 414/4332, respectively. Moreover, the Z-line did not cross the sample size boundary for the risk of acute kidney injury in this population of patients, with a ratio of n = 2561/7569. Therefore, more research is warranted on these outcomes in patients undergoing major surgery.

In contrast, the TSAs showed the robustness of the meta-analysis findings on the length of hospital and ICU stay and the duration of mechanical ventilation, with a ratio of patients recruited/needed of n = 2616/931, n = 2711/1651 and n = 1688/1397, respectively. The role of GDT in reducing the risk of heart failure and pulmonary edema was also robust (n = 1148/1035).

In summary, these further analyses revealed the need for additional research to investigate the impact of GDT on the risk of mortality, myocardial infarction, hypotension and acute kidney injury. However, no additional studies are warranted to validate the well-established role of GDT in the length of hospital and ICU stay and the duration of mechanical ventilation in patients undergoing major surgery.
