# Supplementary material for: To: Goal-directed therapy guided by the FloTrac sensor in major surgery: a systematic review and meta-analysis
Source: Crit Care Sci. 2024 Nov 26;36:e20240168en. doi: 10.62675/2965-2774.20240168-en (PMC11464003; doi:10.62675/2965-2774.20240168-en)
Supplement: Translation

# Translation: Para: Terapia guiada por metas utilizando o sensor FloTrac em cirurgia de grande porte: revisão sistemática e metanálise

**DOI:** 10.62675/2965-2774.20240168-pt

**Authors:** Luigi La Via, Giacomo Cusumano, Christian Zanza, Carmelo Calvagna, Antonino Maniaci

**Affiliations:** 1 Catania Itália Departamento de Anestesia e Cuidados Intensivos, Azienda Ospedaliero Universitaria Policlinico "G. Rodolico - San Marco" - Catania, Itália.; 2 Catania Itália Departamento de Cirurgia e Especialidades Médico-Cirúrgicas, University of Catania - Catania, Itália.; 3 Roma Itália Programa de Residência em Medicina Geriátrica, University of Rome "Tor Vergata" - Roma, Itália.; 4 Pittsburgh Estados Unidos Departamento de Anestesiologia e Medicina Perioperatória, University of Pittsburgh - Pittsburgh, Estados Unidos.; 5 Enna Itália Faculty of Medicine and Surgery, University "Kore" - Enna, Itália.

Ao Editor

Lemos com grande interesse a metanálise de Alves et al.,^(1)^ que destacou os efeitos da terapia guiada por metas (TGM) utilizando o sensor FloTrac em pacientes submetidos a cirurgias de grande porte. Essa metanálise de estudos controlados e randomizados (ECRs) é relevante e confirma um papel possivelmente preferencial na TGM nessa população de pacientes, considerando a melhora significativa nos resultados clínicos, o menor tempo de internação hospitalar e na unidade de terapia intensiva (UTI) e o menor tempo de ventilação mecânica. Esses achados estão alinhados aos da literatura atual, uma vez que o monitoramento hemodinâmico minimamente invasivo demonstrou recentemente potencial para orientar a fluidoterapia tanto na sala de cirurgia quanto na UTI.^(2,3)^

Entretanto, antes de tirar conclusões definitivas sobre o papel da TGM em pacientes submetidos a cirurgias de grande porte, faz-se necessária uma análise da robustez dos achados fornecidos por Alves et al.^(1)^ Portanto, acreditamos que o manuscrito se beneficiaria muito da inclusão de análises sequenciais de ECRs, o que nos permitiria calcular a amostra necessária ("tamanho da informação"), estimar o poder da metanálise nos desfechos investigados, bem como a necessidade de mais pesquisas.

Assim, gostaríamos de oferecer essa contribuição. Importamos os mesmos dados fornecidos pelos autores no programa *Trial Sequential Analysis* (TSA, Copenhagen Trial Unit's TSA Software^®^; Copenhague, Dinamarca). O tamanho da informação foi calculado com base em um risco alfa de 5%, com poder de 80%. A mortalidade estimada foi calculada pela média ponderada dos estudos incluídos. Empregou-se um modelo de efeitos aleatórios, com resultados analisados como risco relativo ou diferença média. Mais detalhes sobre análises sequenciais de ECRs e sua interpretação estão disponíveis em outros artigos.^(4,5)^

As análises sequenciais de ECRs revelaram que as evidências atuais são bastante insuficientes para determinar se a TGM reduz a mortalidade em comparação com os cuidados habituais. Da mesma forma, as razões entre o número de pacientes recrutados e a amostra necessária de risco de infarto do miocárdio e hipotensão foram n = 1.172/5.415 e n = 414/4.332, respectivamente. Além disso, a linha Z não ultrapassou o limite de tamanho da amostra quanto ao risco de lesão renal aguda nessa população de pacientes, com uma relação de n = 2.561/7.569. Assim, são imprescindíveis mais pesquisas sobre esses desfechos em pacientes submetidos a cirurgias de grande porte.

Em contrapartida, as análises sequenciais de ECRs mostraram a robustez dos achados da metanálise no tempo de internação hospitalar e na UTI e na duração da ventilação mecânica, com proporção de pacientes recrutados/necessários de n = 2.616/931, n = 2.711/1.651 e n = 1.688/1.397, respectivamente. O papel da TGM na redução do risco de insuficiência cardíaca e edema pulmonar também foi robusto (n = 1.148/1.035).

Resumidamente, essas novas análises sequenciais de ECRs revelaram a necessidade de mais pesquisas para investigar o impacto da TGM no risco de mortalidade, infarto do miocárdio, hipotensão e lesão renal aguda. No entanto, não há necessidade de mais estudos para validar a função bem estabelecida da TGM no tempo de internação hospitalar e na UTI e na duração da ventilação mecânica em pacientes submetidos a cirurgias de grande porte.
